# How perceived environmental quality influences physical activity in urban green spaces? A moderated mediation model

**DOI:** 10.3389/fpubh.2025.1670171

**Published:** 2025-12-15

**Authors:** Peng Zhang, Yuanzheng Lin, Bin Zhao, Xiujie Ma, Qingyuan Luo, Changxu La

**Affiliations:** 1 College of Physical Education and Sports, Beijing Normal University, Beijing, China; 2 College of Physical Education and Health Science, Yibin University, Yibin, China; 3 School of Wushu, Chengdu Sport University, Chengdu, China; 4 Xi’an Physical Education University, Xi’an, China

**Keywords:** urban green space, physical activity, perceived environmental quality, perceived restorativeness, spatial accessibility, moderated mediation

## Abstract

**Objective:**

This study aims to examine how residents’ perceptions of urban green space environments influence their physical activity levels. Specifically, it investigates the mediating role of perceived restorativeness and the moderating role of spatial accessibility.

**Methods:**

A cross-sectional survey was conducted with 307 residents in Chengdu, China. Structured questionnaires were used to measure perceived environmental quality, perceived restorativeness, spatial accessibility, and physical activity level. Confirmatory factor analysis (CFA) and model fit assessments were performed in AMOS 24.0 to evaluate the reliability and validity of the measurement model. Mediation and moderation analyses were subsequently conducted in SPSS 26.0 using the PROCESS macro to test the indirect and interaction effects.

**Results:**

Perceived environmental quality was positively associated with residents’ physical activity levels (*β* = 0.707, *p* < 0.001). Perceived restorativeness partially mediated this relationship (*β* = 0.229, *p* < 0.001). Moreover, spatial accessibility significantly moderated the association (*β* = 0.185, *p* < 0.001), with a stronger relationship observed under higher accessibility conditions.

**Conclusion:**

This study highlights the roles of psychological and spatial factors in understanding urban green space–related physical activity. Enhancing environmental quality, accessibility, and restorative characteristics of green spaces may help create conditions that are more conducive to residents’ engagement in physical activity and overall well-being.

## 1 Introduction

With the accelerating pace of urbanization, the spatial and temporal availability of daily physical activity for urban residents has become increasingly constrained. Rising population density, prolonged commuting times, and shrinking public spaces have collectively imposed multifaceted barriers to individuals’ engagement in physical exercise. Although a wealth of research has consistently demonstrated the significant benefits of physical activity in promoting both physical and mental health—including preventing chronic diseases, alleviating psychological stress, and fostering social connectedness (1–3)—urban dwellers continue to fall short in maintaining adequate levels of exercise. This persistent insufficiency in physical activity has emerged as a critical public health concern.

Urban green spaces, as one of the most accessible and multifunctional environments for physical activity, play a vital role in supporting exercise participation by offering natural settings that promote emotional relief and bodily movement (4). Recent studies have indicated that high-quality urban green spaces are positively associated with increased exercise frequency and higher levels of physical activity among residents (5, 6). However, substantial disparities remain across different green spaces in terms of environmental quality (e.g., facility completeness, cleanliness, safety), spatial distribution (e.g., proximity), and temporal accessibility (e.g., lighting and opening hours), all of which significantly influence individuals’ place selection and exercise behaviors (7–9).

Furthermore, environmental psychology suggests that individuals’ subjective evaluations of the environment shape not only their behavioral intentions but also their emotional restoration (10), thereby enhancing their motivation and likelihood to engage in physical activity within that environment.

Against this backdrop, the present study focuses on urban green space-based physical activity and proposes a theoretical framework based on a perception–psychological–behavioral pathway model. Specifically, we conceptualize perceived environmental quality as the independent variable, perceived restorativeness as the mediating variable, and physical activity level as the dependent variable. Additionally, spatial accessibility is introduced as a moderating factor to examine how residents’ subjective perceptions of green spaces influence their exercise behaviors through psychological mechanisms, and how this process may vary under different levels of accessibility.

## 2 Literature review and research hypotheses

### 2.1 The direct effect of perceived environmental quality on physical activity level

Perceived environmental quality refers to individuals’ subjective assessment of environmental attributes within a specific space, encompassing factors such as the completeness of facilities, cleanliness and comfort, safety, greenness, and visual aesthetics (11). According to the Social Ecological Model, environmental influences on behavior operate not only through objective conditions but also through individuals’ subjective perceptions, which play a critical role in behavioral decision-making (12).

In recent years, an increasing number of studies have examined how environmental characteristics of urban green spaces affect residents’ physical activity behavior (13–15). First, in terms of facility adequacy, green spaces equipped with functional infrastructure—such as fitness equipment, walking paths, and lighting systems—have been shown to stimulate residents’ willingness to engage in physical activity and increase participation frequency (16). The availability of exercise-friendly infrastructure lowers the entry threshold, making it more convenient for individuals to incorporate green spaces into their daily exercise routines (17).

Second, with regard to cleanliness and comfort, environments that are tidy, quiet, and well-vegetated have been associated with higher satisfaction levels, longer stays, and greater physical activity engagement (18). Sensory elements such as visual and olfactory comfort contribute to more pleasant exercise experiences (19). Third, safety is another critical dimension—green spaces with sufficient lighting, surveillance infrastructure, and moderate foot traffic are especially valued by those who exercise at night. A heightened sense of safety can significantly enhance participation rates, particularly among women and older adults (20).

In summary, high-quality urban green spaces provide a more supportive and satisfying context for physical activity, thereby enhancing residents’ overall exercise levels. Based on the aforementioned theoretical and empirical insights, the following hypothesis is proposed:

*H1*: Perceived environmental quality of urban green spaces is positively associated with residents’ physical activity levels.

### 2.2 The mediating role of perceived restorativeness

Perceived restorativeness refers to an individual’s subjective sense of psychological recovery experienced within a given environment. This typically manifests as attention restoration, emotional relaxation, relief from daily stressors, and a sense of inner pleasure (21). According to Attention Restoration Theory (ART), natural or nature-like environments promote “soft fascination” and a sense of “being away,” which facilitate the replenishment of cognitive resources depleted by prolonged directed attention in daily life, thereby enhancing mood and behavioral intentions (10).

Urban green spaces, as prototypical “high-restorative” environments, are widely recognized for their potential to support psychological recovery (22). Environmental quality is considered a key determinant of residents’ restorative experiences in such settings (23, 24). Studies have shown that features such as tranquility, greenery, and detachment from urban noise contribute to higher levels of perceived restorativeness (19, 25). When individuals perceive urban green spaces as high in environmental quality, they are more likely to experience feelings of relaxation, escape, and psychological renewal, which in turn enhance their motivation and willingness to engage in physical activity (26).

In the context of high-pressure urban life, perceived restorativeness influences not only short-term emotional states but may also serve as an internal driver of sustained physical activity. For example, research has found that individuals prefer to exercise in environments that offer restorative experiences and report higher levels of exercise adherence and satisfaction when doing so (27). Therefore, it is plausible that perceived restorativeness mediates the relationship between perceived environmental quality and residents’ physical activity levels.

Based on the above theoretical and empirical foundations, the following hypothesis is proposed:

*H2*: Perceived restorativeness mediates the relationship between perceived environmental quality and residents’ physical activity levels in urban green spaces.

### 2.3 The moderating role of spatial accessibility

Spatial accessibility refers to the ease with which individuals can reach a target destination from their place of residence, within given temporal, spatial, and transportation constraints. It is commonly operationalized through perceived distance, walking time, or transportation convenience (28). In the context of urban public health and physical activity research, spatial accessibility is widely recognized as a critical environmental determinant of residents’ physical activity levels (29). Exercise settings with high accessibility are more likely to be integrated into daily travel routines, thereby increasing actual utilization and activity frequency (30, 31).

According to the Social Ecological Model (32), individual behaviors are shaped not only by internal psychological factors but also by external environmental resources. Even if residents perceive the environmental quality of a green space to be high, low geographic accessibility—such as inconvenient transportation, long travel times, or mobility barriers—may prevent this positive perception from translating into actual behavior. In other words, spatial accessibility may serve as a crucial moderator in the relationship between perceived environmental quality and physical activity engagement.

Empirical evidence supports this interaction mechanism. For instance, McCormack et al. (17) found that no matter how well-equipped a green space is, if it is located far from residential areas, its usage and associated physical activity levels are significantly reduced. Similarly, Giles-Corti et al. (33) reported that each additional 5 min of walking distance decreases the likelihood of residents engaging in moderate-intensity exercise in parks by 17%. The influence of accessibility is especially pronounced among older adults, for whom mobility constraints are more limiting (34).

Thus, spatial accessibility may shape the extent to which residents’ positive perceptions of green space quality are associated with actual physical activity. Under conditions of high accessibility, these positive perceptions are more likely to translate into frequent and sustained engagement in physical activity; conversely, when accessibility is low, even favorable perceptions may be insufficient to overcome logistical barriers.

Based on this rationale, the following hypothesis is proposed:

*H3*: Spatial accessibility moderates the relationship between perceived environmental quality and physical activity levels, such that the relationship is stronger under conditions of higher spatial accessibility.

### 2.4 Hypotheses and conceptual model

**Figure 1:**
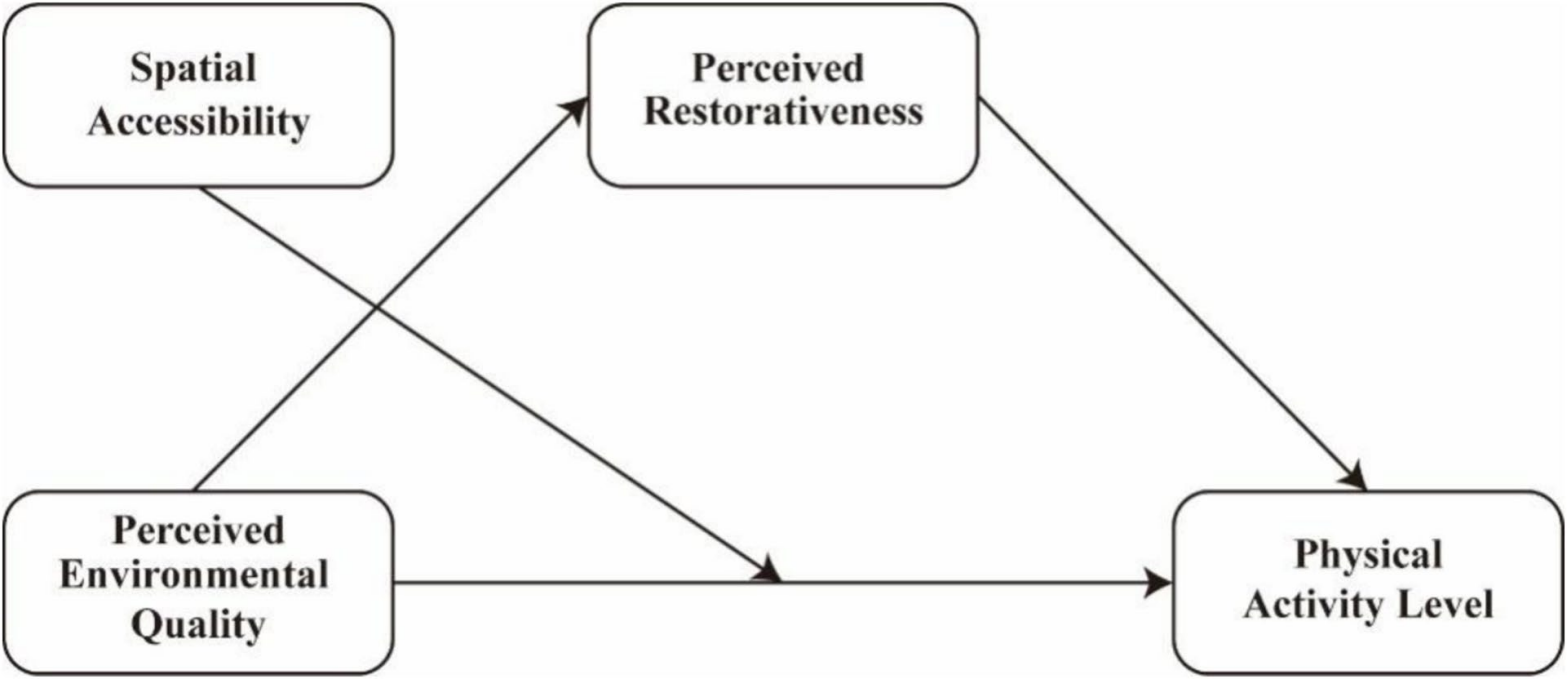
The hypothetical model.

Based on the theoretical foundations discussed in the previous sections, this study constructs a conceptual model to explore how perceived environmental quality influences urban residents’ physical activity level through psychological and spatial mechanisms. The model posits that perceived environmental quality has a direct effect on physical activity level (H1). At the same time, perceived restorativeness functions as a psychological mechanism that translates environmental perceptions into motivational and behavioral outcomes, thereby mediating this relationship (H2). Furthermore, spatial accessibility is hypothesized to moderate the direct paths (H3). The proposed research model and hypotheses are depicted in Figure 1.

## 3 Materials and methods

### 3.1 Participants and procedure

This study was conducted in two representative urban green spaces in Chengdu, the capital city of Sichuan Province, China: Park A and Green Plaza B. Park A was selected for its well-maintained facilities and high perceived environmental quality, whereas Plaza B represented a contrasting environment characterized by relatively outdated infrastructure and lower environmental standards. The contrasting features of these two sites provided a meaningful spatial comparison and enhanced the contextual representativeness of the study. Both sites are located in densely populated urban districts with high levels of green space utilization, making them suitable settings for examining the associations between environmental perception and physical activity among urban residents.

Participants were individuals who regularly engaged in physical activity within the two designated green spaces. A combination of convenience sampling and on-site questionnaire distribution was employed for data collection. To ensure sample diversity and minimize temporal bias, data were collected across five separate sessions at both sites, covering key activity periods including early morning, evening, and nighttime hours.

Inclusion criteria required participants to (1) be over 18 years old; (2) have engaged in physical activity within the past 6 months, at least once per week; and (3) self-report normal physical and mental health conditions sufficient for independent participation in exercise. Individuals reporting serious health problems or mobility restrictions were not included, to ensure data reliability and participant safety.

In total, 320 questionnaires were distributed, and 307 valid responses were retained after excluding incomplete or logically inconsistent entries, resulting in a valid response rate of 95.9%. During data collection, participants were fully informed about the study’s purpose, voluntary participation, and the anonymity and confidentiality of their responses. All participants provided informed consent prior to completing the survey. No sensitive personal information was collected. This study was conducted in accordance with the ethical standards for social science research and was approved by the Ethics Committee of Chengdu Sport University.

### 3.2 Instruments

#### 3.2.1 Perceived environmental quality

Drawing on environmental psychology and previous studies on urban green space quality perception (11, 35, 36), this study operationally defined perceived environmental quality as residents’ subjective evaluation of the environmental conditions of their regular exercise venues. This construct encompasses three dimensions: facility adequacy, environmental cleanliness and comfort, and perceived safety. Facility adequacy assesses the quantity, functionality, and lighting conditions of available exercise facilities. Environmental cleanliness and comfort reflect perceptions of spatial tidiness, vegetation coverage, and noise levels. Perceived safety captures the subjective sense of safety when engaging in physical activity within the green space. Each dimension was measured using 3, 3, and 2 items respectively, resulting in a total of 8 items. All items were rated on a 5-point Likert scale (1 = strongly disagree, 5 = strongly agree). In the current study, the scale demonstrated good internal consistency, with a Cronbach’s alpha of 0.889.

#### 3.2.2 Perceived restorativeness

Grounded in Attention Restoration Theory (10) and drawing on the Perceived Restorativeness Scale (PRS) developed by Hartig et al. (21), this study conceptualized perceived restorativeness as comprising five dimensions: being-away, fascination, compatibility, coherence, and extent. Being-away captures the extent to which individuals feel removed from daily stressors and routine demands. Fascination refers to the environment’s capacity to effortlessly attract attention. Compatibility reflects the degree to which the environment supports the individual’s intended activities and goals. Coherence assesses whether the environment is easy to comprehend, orderly, and harmonious. Extent measures the perceived spaciousness and the sense of immersion or psychological expansion within the space. Each dimension included two items, totaling 10 items overall. All items were rated on a 5-point Likert scale (1 = strongly disagree, 5 = strongly agree). In this study, the scale demonstrated excellent internal consistency, with a Cronbach’s alpha of 0.903.

#### 3.2.3 Spatial accessibility

Drawing on the frameworks proposed by Sallis et al. (12), Zhang and Li (37), and McCormack and Shiell (38), this study conceptualized spatial accessibility as comprising three interrelated dimensions: time cost, perceived distance, and accessibility barriers. Time cost refers to individuals’ subjective assessment of the time required to reach their exercise destination. Perceived distance captures the spatial proximity of the exercise location as perceived by the respondent. Accessibility barriers measure potential impediments to access, such as transportation limitations, route difficulty, or safety concerns. All items were rated on a 5-point Likert scale (1 = strongly disagree, 5 = strongly agree). In this study, the Cronbach’s alpha for the spatial accessibility scale was 0.932, indicating high internal consistency reliability.

#### 3.2.4 Physical activity level

This study measured the physical activity level of urban residents in green spaces using the Physical Activity Rating Scale (PARS-3), developed by Liang (39). The scale has demonstrated good reliability and validity in assessing individual participation in physical activity and has been widely adopted in related research across China. The PARS-3 quantifies physical activity based on three components: duration, frequency, and intensity of exercise. Duration is scored on a scale from 0 to 4, while both frequency and intensity are scored from 1 to 5. The overall physical activity level is calculated using the following formula: Activity Level = Duration × Frequency × Intensity. The resulting score ranges from 0 to 100, with higher scores indicating greater participation in physical activity. In this study, the Cronbach’s alpha coefficient for the physical activity scale was 0.868, reflecting good internal consistency reliability.

### 3.3 Control variables

Given that physical activity behavior is influenced by multiple factors beyond the main explanatory variables, this study includes a set of demographic and socio-economic characteristics as control variables. Specifically, gender, age, education level, occupation, and years of residence are included to account for potential confounding effects in the analysis.

### 3.4 Analysis

Data analysis in this study was conducted using AMOS 24.0, SPSS 26.0, and Hayes’ PROCESS macro 3.4. To assess potential common method bias (CMB), the Common Latent Factor (CLF) approach was employed. A latent method factor was added to the confirmatory factor analysis (CFA) model, allowing all observed indicators to load simultaneously on both their theoretical constructs and the CLF. Following the recommendations of Podsakoff et al. (40), model fit indices were compared between the original and the CLF-adjusted models. If the changes in GFI, CFI, TLI, and RMSEA indices are less than 0.01, then CMB is not considered a serious issue, CMB is considered not a serious concern (41). In this study, the differences in model fit indices were minimal (ΔCFI < 0.01, ΔRMSEA < 0.01), suggesting that common method bias was not a major issue.

Descriptive statistics, reliability tests, and correlation analyses were conducted using SPSS 26.0. Confirmatory factor analysis (CFA) and model fit assessments were performed in AMOS 24.0 to evaluate the reliability and validity of the measurement model. Pearson correlation coefficients were used to assess the bivariate relationships among key variables.

To test the hypothesized mediating and moderating effects, the bootstrapping method (5,000 resamples) was applied using Hayes’ PROCESS macro (42). Specifically, Model 4 was used to test the mediating effect of perceived restorativeness, and Model 5 was used to examine the moderating effect of spatial accessibility. In addition, simple slope analysis was conducted to visualize and interpret the interaction effect of the moderator on the relationship between perceived environmental quality and physical activity level.

## 4 Results

### 4.1 Descriptive statistics and correlations among the main study variables

The sample of this study consisted of 307 participants. As shown in Figure 1, this study investigated the participants’ gender, age, educational level, occupation, and length of residence in the local area. The respondents ranged in age from 18 to 65 years, with 52.44% identifying as male and 47.56% as female. Most of the respondents were 45 years old or above, held a bachelor’s degree or higher, and were engaged in various occupations, including company employees, self - employed individuals, and retirees. 55.05% of the participants had lived locally for more than 5 years (see Table 1).

**Table 1:** Demographic characteristics of the samples (*N* = 307).

Variable	Category	Frequency (*n*)	Percentage (%)
Gender	Male	161	52.44%
	Female	146	47.56%
Age	18–29	64	20.85%
	30–44	58	18.89%
	45–59	87	28.34%
	60 and above	98	31.92%
Education Level	High school or below	41	13.36%
	Junior college	92	29.97%
	Bachelor’s degree	124	40.39%
	Master’s or above	50	16.28%
Occupation	Company employee	106	34.53%
	Self-employed	45	14.65%
	Government/institutional	37	12.05%
	Student	26	8.47%
	Retired	71	23.13%
	Other	22	7.17%
Years of Residence	Less than 1 year	20	6.52%
	1–3 years	51	16.61%
	3–5 years	67	21.82%
	More than 5 years	169	55.05%

Table 2 presents the means, standard deviations, and Pearson correlation coefficients among the primary study variables. The mean scores ranged from 2.93 to 3.13, with standard deviations between 0.581 and 0.812, reflecting moderate central tendencies and acceptable levels of dispersion. All variables were significantly correlated in the expected directions. Importantly, no correlation coefficient exceeded the commonly accepted multicollinearity threshold of 0.85, indicating no multicollinearity concerns.

**Table 2:** Descriptive statistics and correlations among primary variables.

Variable	M	SD	1	2	3	4
1 Perceived environmental quality	2.93	0.668	1			
2 Perceived restorativeness	2.97	0.581	0.622^**^	1		
3 Spatial accessibility	3.13	0.704	0.542^**^	0.515^**^	1	
4 Physical activity level	3.04	0.812	0.770^**^	0.665^**^	0.773^**^	1

*p < 0.05; **p < 0.01 (two-tailed).

### 4.2 The test of reliability and validity

To assess the reliability and convergent validity of the constructs, composite reliability (CR) and average variance extracted (AVE) were calculated for each latent variable. As shown in Table 3, the CR values for the four core constructs ranged from 0.879 to 0.933, while the AVE values ranged from 0.639 to 0.708. According to Chin (43), CR values above 0.70 and AVE values above 0.50 indicate acceptable levels of internal consistency and convergent validity.

**Table 3:** Validity and reliability tests of the questionnaires.

Variable	CR	AVE
Perceived environmental quality	0.879	0.708
Perceived restorativeness	0.898	0.639
Spatial accessibility	0.933	0.667
Physical activity level	0.869	0.690

CR, composite reliability; AVE, average variance extracted.

In addition, several model fit indices were evaluated to assess the overall goodness of fit, including *χ^2^/df*, GFI, RFI, CFI, NFI, IFI, SRMR and RMSEA. As presented in Table 4, all fit indices exceeded the recommended threshold of 0.90—specifically, GFI = 0.931, RFI = 0.944, CFI = 0.981, NFI = 0.952, and IFI = 0.982—indicating an excellent model fit. The SRMR value was 0.030, RMSEA value was 0.044, well below the conventional cutoff of 0.08, suggesting a close and acceptable fit between the hypothesized model and the observed data (44).

**Table 4:** Model fit indices for the measurement model.

Fit indices	*χ2/df*	GFI	RFI	CFI	NFI	IFI	SRMR	RMSEA
Indices	1.605	0.931	0.944	0.981	0.952	0.982	0.030	0.044

GFI, goodness of fit index; RFI, relative fit index; CFI, comparative fit index; NFI, normed fit index; IFI, incremental fit index; RMSEA, root mean square error of approximation.

### 4.3 The mediation model analysis

**Figure 2:**
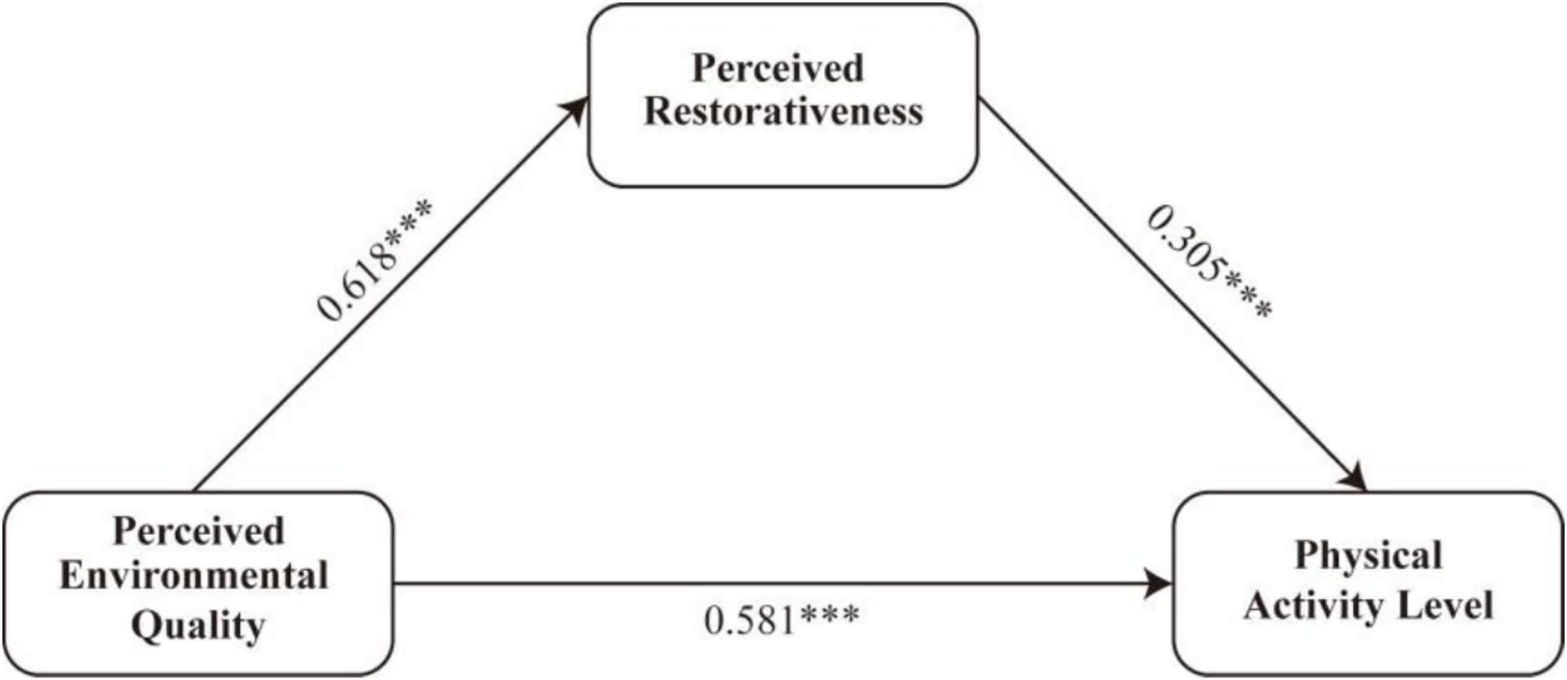
Standardized path coefficients of the mediation model. ***p < 0.001.

This study employed regression analysis and a bootstrapping approach to examine the mediating role of perceived restorativeness in the relationship between perceived environmental quality and physical activity level. The standardized path coefficients of the mediation model are shown in Figure 2.

As shown in Table 5, the first-step regression analysis revealed that perceived environmental quality significantly predicted perceived restorativeness (*β* = 0.537, *p* < 0.001). In the second-step regression, both perceived environmental quality (*β* = 0.707, *p* < 0.001) and perceived restorativeness (*β* = 0.426, *p* < 0.001) showed significant positive effects on physical activity level.

**Table 5:** Regression analysis of the mediation model.

Predictors	Step 1 (Perceived Restorativeness)			Step 2 (Physical Activity Level)		
	*β*	SE	t	*β*	SE	t
Constant	1.469	0.200	7.316***	−0.517	0.230	−2.241
Gender	0.044	0.052	0.849	0.026	0.056	0.468
Age	−0.010	0.023	−0.428	−0.011	0.025	−0.432
Education level	−0.001	0.028	−0.048	0.038	0.030	1.237
Occupation	−0.001	0.015	−0.039	0.003	0.015	0.231
Years of residence	−0.032	0.027	−1.164	0.030	0.029	1.026
Perceived environmental quality	0.537	0.039	13.670***	0.707	0.053	13.345***
Perceived restorativeness				0.426	0.061	6.976***
*R* ^2^	0.392			0.653		
*F*	32.254***			80.432***		

***p < 0.001.

To further validate the mediation effect, a nonparametric bootstrap analysis (5,000 resamples) was conducted using PROCESS macro (Model 4). As shown in Table 6, the total effect of perceived environmental quality on physical activity level was significant [*β* = 0.936, 95% CI (0.849, 1.024)]. The direct effect remained significant [*β* = 0.707, 95% CI (0.603, 0.811)], accounting for 75.53% of the total effect. The indirect effect through perceived restorativeness was also significant [*β* = 0.229, 95% CI (0.156, 0.305)], explaining 24.47% of the total effect.

**Table 6:** Bootstrapping results of the mediation model.

	Effect	SE	95% CI	Ratio to total effect
Direct effect	0.707	0.052	(0.603, 0.811)	75.53%
Indirect effect	0.229	0.038	(0.156, 0.305)	24.47%
Total effect	0.936	0.044	(0.849, 1.024)	—

Taken together, these findings provide empirical support for a partial mediation model, indicating that perceived environmental quality is not only directly associated with higher physical activity levels but also indirectly related to them through enhanced perceived restorativeness. Thus, both Hypothesis 1 and Hypothesis 2 were supported.

### 4.4 The moderating model analysis

As presented in Table 7, the interaction term between perceived environmental quality and spatial accessibility (Perceived Environmental Quality × Spatial Accessibility) was found to exert a significant positive effect on physical activity level (*β* = 0.185, *p* < 0.001), thereby providing empirical support for a significant moderating effect and confirming Hypothesis 3. This finding suggests that the magnitude of the impact of perceived environmental quality on physical activity is contingent upon the level of spatial accessibility. Specifically, the positive association between environmental quality and physical activity is strengthened under conditions of high accessibility, whereas this relationship becomes attenuated when accessibility is low.

**Table 7:** Regression results for the moderation model.

Predictors	Outcome variable: physical activity level			
	*β*	SE	*t*	Bootstrap 95% CI (LL, UL)
Constant	2.339	0.197	12.160***	(2.010, 2.787)
Gender	−0.029	0.041	−0.693	(−0.111, 0.053)
Age	0.006	0.018	0.321	(−0.030, 0.042)
Education level	0.002	0.022	−0.114	(−0.047, 0.042)
Occupation	0.002	0.011	0.153	(−0.021, 0.025)
Years of residence	0.011	0.022	0.499	(−0.032,0.054)
Perceived environmental quality	0.580	0.044	13.149***	(0.493, 0.667)
Spatial accessibility	0.583	0.037	15.489***	(0.509, 0.657)
Perceived environmental quality × spatial accessibility	0.185	0.034	5.459***	(0.118, 0.252)
*R* ^2^	0.809			
*F*	140.148***			

***p < 0.001; Bootstrap 95% CI based on 5,000 samples.

As shown in Table 8, perceived environmental quality significantly predicted physical activity level at all levels of spatial accessibility. The simple slope was weakest at low spatial accessibility (*β* = 0.449, *p* < 0.001), moderate at the mean level (*β* = 0.580, *p* < 0.001), and strongest at high spatial accessibility (*β* = 0.710, *p* < 0.001). These results indicate that the positive effect of perceived environmental quality on physical activity becomes progressively stronger as spatial accessibility increases.

**Table 8:** Simple slopes of perceived environmental quality predicting physical activity level at different levels of spatial accessibility.

Perceived stress	Effect	SE	*t*	*p*	95% CI (LL, UL)
Low (−1 SD)	0.449	0.043	10.470	<0.001	(0.365, 0.534)
Mean	0.580	0.044	13.149	<0.001	(0.493, 0.667)
High (+1 SD)	0.710	0.565	12.581	<0.001	(0.599, 0.822)

**Figure 3:**
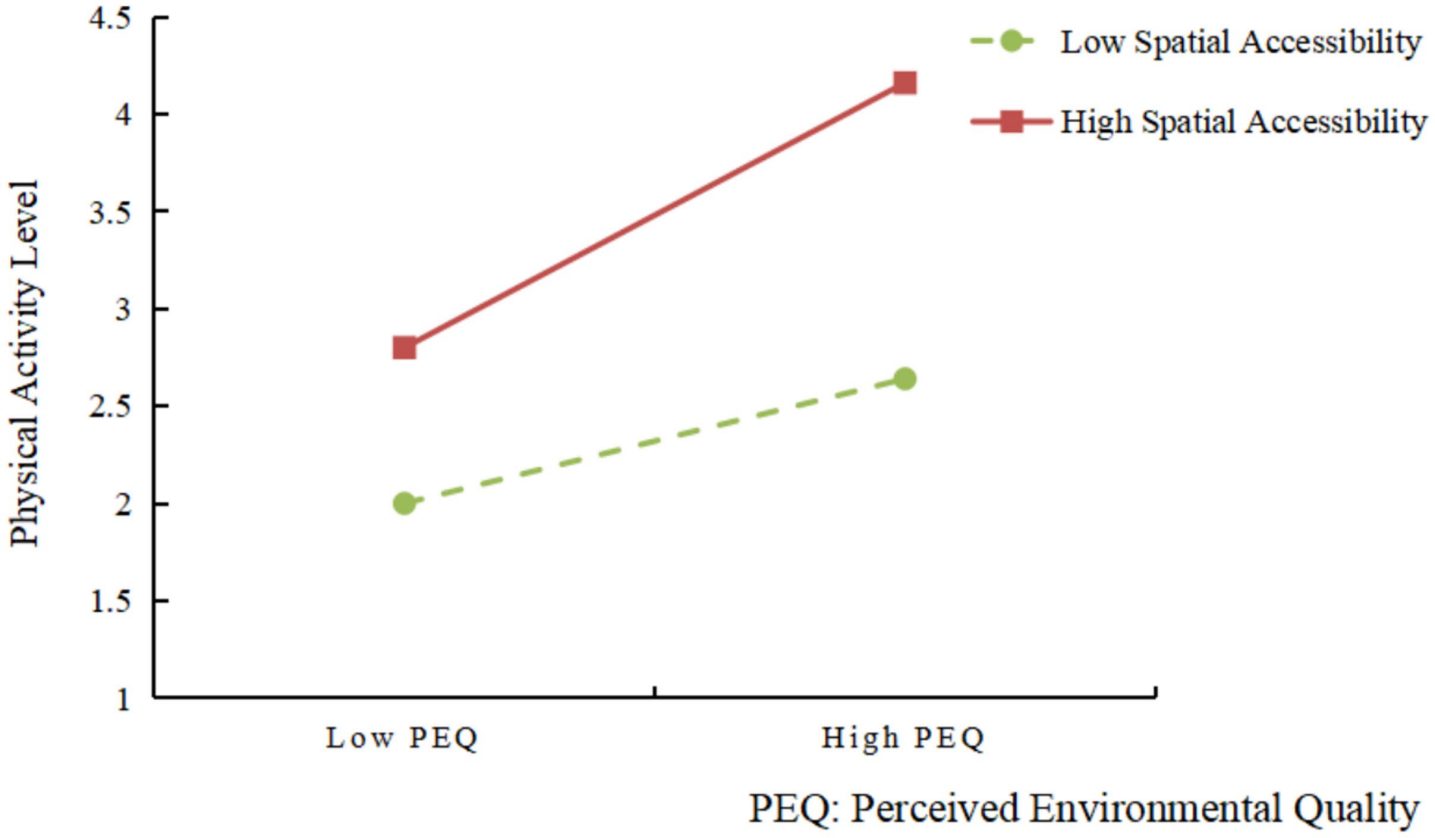
Interaction between perceived environmental quality and spatial accessibility.

As illustrated in Figure 3, the interaction pattern is visually consistent with the simple slopes reported in Table 8. When spatial accessibility is low, increases in perceived environmental quality are associated with only a modest rise in physical activity level, reflected in the relatively flat slope. In contrast, under high spatial accessibility, the slope becomes substantially steeper, indicating that improvements in environmental quality lead to markedly greater increases in physical activity. This divergence between the two lines further confirms that spatial accessibility strengthens the positive impact of perceived environmental quality on physical activity, amplifying the behavioral benefits of high-quality urban green spaces.

## 5 Discussion

### 5.1 The direct effect of perceived environmental quality on physical activity level

This study found that perceived environmental quality was significantly and positively associated with urban residents’ physical activity levels. This finding is highly consistent with prior research, which has repeatedly reported that high-quality environments are linked to greater willingness and frequency to engage in physical activity (6, 45, 46). Specifically, when individuals perceive their exercise environments as clean, well-equipped with fitness facilities, aesthetically pleasing, and safe, they are more likely to maintain consistent physical activity routines.

Similar patterns have been reported in cross-cultural studies. For instance, research conducted in European and North American cities has also found that perceived environmental quality and accessibility are positively associated with residents’ physical activity levels, largely through enhanced restorative experiences (36, 47). However, cultural differences may shape how individuals interpret and utilize urban green spaces. In East Asian contexts, for example, collective exercise forms such as square dancing or tai chi are more prevalent (48, 49), suggesting that social and cultural norms may further mediate the influence of environmental perception on activity behaviors.

The social ecological model posits that health behaviors are shaped not only by internal motivations but also by structural environmental conditions (12). In urban contexts, perceived environmental comfort, safety, and convenience may help reduce psychological barriers to exercise, enhance motivation, and ultimately increase actual physical activity levels.

Compared to previous studies, the present research deepens the conceptualization of environmental quality by emphasizing subjective perceptions in addition to objective conditions. It operationalizes perceived environmental quality across multiple dimensions—including facility provision, natural characteristics, and cleanliness and safety—thus enriching our understanding of the relationship between environmental attributes and health behaviors. The findings suggest that residents’ subjective evaluations may be more closely related to behavioral choices than objective metrics such as green space area.

From a practical standpoint, urban planners and policymakers seeking to promote healthy lifestyles should prioritize not only the quantity of green space but also its quality and user experience. In particular, psychologically restorative and behaviorally supportive design features—such as adequate lighting, clear signage, accessible pathways, water features, and shaded resting areas—can enhance users’ comfort, safety, and enjoyment, thereby encouraging more frequent participation in physical activity. Integrating these design principles into local planning standards and community park renewal projects would help translate environmental quality improvements into tangible public health benefits.

### 5.2 The mediating role of perceived restorativeness

This study further confirmed the mediating role of perceived restorativeness in the relationship between perceived environmental quality and physical activity level. Consistent with prior findings (50, 51), the results suggest that environmental perceptions may relate to residents’ physical activity not only directly but also indirectly through psychological restoration. This finding enriches the theoretical understanding of the perception–psychology–behavior pathway by highlighting the potential cognitive and emotional processes that individuals engage in when experiencing environmental stimuli.

Specifically, when urban green spaces are perceived as pleasant, well-ordered, and environmentally friendly, they tend to be associated with restorative experiences such as attention recovery, emotional relaxation, a sense of escape, and self-reflection (52, 53). These positive psychological responses may help alleviate mental fatigue and are linked to more favorable emotional states, which in turn may be associated with greater motivation for physical activity. This mechanism is consistent with the propositions of Attention Restoration Theory (ART) and Stress Reduction Theory (SRT), both of which indicate that restorative experiences in natural environments are related to beneficial health-related behaviors (54).

Moreover, unlike many previous studies focusing solely on objective environmental factors, this research introduces perceived restorativeness as a key psychological mediator, providing a more comprehensive view of the environmental–psychological–behavioral chain. The results emphasize that urban green spaces function not only as physical venues for activity but also as settings that relate to internal psychological states. This underscores the importance of subjective perception and psychological mechanisms in models of public health behavior.

From a practical standpoint, the findings suggest that urban space governance should go beyond physical functionality to consider the psychological support value of environments. Urban green space design can be improved by incorporating restorative features such as quiet zones, meditation areas, water elements, and diverse landscapes, combined with quantifiable planning indicators—such as visual openness, noise reduction, and perceived safety indices—to evaluate “psychological friendliness.” Integrating these indicators into design guidelines or local planning standards would provide an operational framework for enhancing residents’ restorative experiences and, consequently, their engagement in physical activity.

### 5.3 The moderating role of spatial accessibility

This study found that spatial accessibility significantly moderates the association between perceived environmental quality and physical activity level. Specifically, when residents perceive a green space as highly accessible, the positive association between environmental quality and physical activity appears stronger. Conversely, when spatial accessibility is low, even high-quality green spaces show a weaker association with residents’ engagement in physical activity—a finding consistent with prior research (55, 56).

This finding suggests that the convenience of access, ease of transportation, and time required to reach a green space may be related to residents’ frequency and likelihood of use. This moderating pattern aligns with the Time–Space Constraint Theory, which posits that individual behaviors are shaped not only by internal intentions but also by external spatial structures and accessibility conditions (57). Thus, even when individuals hold favorable perceptions of a green space, low accessibility—such as long distances, inconvenient transportation, or unsafe routes—may limit the extent to which these perceptions are reflected in actual activity patterns.

Furthermore, spatial accessibility functions as a structural opportunity factor that influences whether individuals’ positive perceptions and intentions are translated into behavioral engagement (30). In high-density, fast-paced urban environments, time and spatial constraints may exacerbate inequalities in opportunities for physical activity. The moderation results highlight that the relationship between environmental perceptions and behavior depends on the degree to which spatial structures support such perceptions.

From a practical perspective, improving the utilization of urban green spaces requires not only enhancing environmental quality but also quantitatively optimizing spatial accessibility. Accessibility assessment can be integrated into urban planning through tools such as GIS-based network analysis, 5- or 10-min walking catchment mapping, and public transport service coverage ratios to identify underserved areas. Planning measures—such as developing continuous pedestrian and cycling corridors, constructing barrier-free routes, and allocating small-scale community parks near residential clusters, schools, and workplaces—can reduce travel time and spatial inequality, thereby facilitating more equitable participation in physical activity.

### 5.4 Limitations and suggestions

Although this study constructed a systematic theoretical model to examine the relationships among perceived environmental quality, perceived restorativeness, spatial accessibility, and physical activity level—and empirically validated several key pathways—there are still some limitations that should be acknowledged and addressed in future research:

First, this study employed a cross-sectional questionnaire design, which, while useful for identifying associations among variables, limits the ability to infer causal relationships. The directionality of these relationships cannot be fully established, and it is also possible that individuals who engage more frequently in physical activity develop more positive perceptions of their environments. Future research should consider adopting longitudinal or experimental designs to verify temporal ordering and enhance causal inference.

Second, the sample was limited to users of specific urban green spaces within a single city, which introduces spatial constraints that may restrict the generalizability of the findings. Exercise behavior patterns may differ across cities, community types, and socio-economic groups. Therefore, future studies should broaden the sampling scope and adopt multi-site or stratified sampling approaches to include populations from diverse urban contexts and social backgrounds, thereby enhancing the external validity and practical applicability of the results.

Third, although major sociodemographic variables were controlled in the analyses, other potential confounding factors—such as individual health status, social support networks, and exercise self-efficacy—were not accounted for. These factors may also influence physical activity behavior. Future studies are encouraged to expand the scope of control variables to better isolate the effects of the primary predictors.

Fourth, the measures used in this study primarily relied on self-reported scales, which may be subject to social desirability bias, recall bias, and other subjective inaccuracies. To improve data objectivity and precision, future research could incorporate behavioral tracking data (e.g., from wearable fitness devices) and spatial data (e.g., GIS-based analyses) to provide a more comprehensive understanding of residents’ actual physical activity patterns.

Finally, the analytical model adopted in this study was based on linear assumptions and did not examine potential nonlinear relationships or higher-order interaction effects among variables (e.g., between environmental quality and accessibility). Although this approach was appropriate for the theoretical framework and sample size of the current study, future research could explore nonlinear or multilevel modeling strategies to capture the complex and dynamic relationships between environmental perceptions and physical activity behaviors in real-world contexts.

## 6 Conclusion

This study conducted an empirical investigation into the relationship between residents’ perceptions of urban green spaces and their physical activity behavior, with particular attention to the mediating role of perceived restorativeness and the moderating role of spatial accessibility. Based on survey data from 307 urban residents, the following key conclusions were drawn:

First, perceived environmental quality significantly and positively predicted physical activity level. Residents who perceived their local green spaces as clean, safe, well-equipped, and aesthetically pleasing were more likely to engage in frequent and intensive physical activity, confirming the facilitating role of high-quality environments in promoting health behaviors.

Second, perceived restorativeness served as a mediator between environmental perception and exercise behavior. High-quality green spaces not only directly encouraged physical activity but also indirectly motivated residents through enhanced psychological restoration experiences—such as attention recovery, emotional relaxation, and escape from daily stress—thereby increasing their overall physical activity levels.

Third, spatial accessibility moderated the relationship between perceived environmental quality and physical activity. Even when residents had positive evaluations of green space quality, low accessibility could reduce their motivation to engage in physical activity. Conversely, green spaces with high accessibility were more likely to be translated into actual use, amplifying the behavioral effects of environmental perception.

In summary, this study constructed and validated a multi-pathway model that integrates subjective environmental perception, psychological mechanisms, and behavioral outcomes. The findings contribute to the theoretical framework of health geography and urban behavior studies, while also providing practical insights for improving the utilization of urban green spaces and promoting public health.

## Data Availability

The original contributions presented in the study are included in the article/supplementary material, further inquiries can be directed to the corresponding author.
